# The future of lung cancer surgery lies between innovations: synergy across the perioperative ERATS pathway

**DOI:** 10.3389/fonc.2026.1919467

**Published:** 2026-08-04

**Authors:** Lars Geenen, Aimée J. P. M. Franssen, Koen C. H. A. Verkoulen, Geertruid M. H. Marres, Carlijn T. I. de Betue, Amir H. Sadeghi, Ghada M. M. Shahin, Gaston H. N. M. Duijsens, Louisa N. Spaans, Frank J. C. van den Broek, Karel W. E. Hulsewé, Yvonne L. J. Vissers, Erik R. de Loos

**Affiliations:** 1 Department of Surgery, Division of Thoracic Surgery, Zuyderland Medical Center, Heerlen, Netherlands; 2 Department of Thoracic Surgery, Lung Cancer Centre, Albert Schweitzer Hospital, Dordrecht, Netherlands; 3 Department of Cardiothoracic Surgery, Radboud University Medical Center, Nijmegen, Netherlands; 4 Department of Cardiothoracic Surgery, University Medical Center Utrecht, Utrecht, Netherlands; 5 Department of Anesthesiology, Zuyderland Medical Center, Heerlen and Sittard-Geleen, Netherlands; 6 Department of Surgery, Máxima Medical Center, Veldhoven, Netherlands

**Keywords:** anesthaesia techniques, RATS, prehabilitation, drain management, lung cancer surgery, 3D imaging

## Abstract

Surgical resection remains the treatment of choice for early-stage lung cancer. Over the past few decades, enhanced recovery after thoracic surgery (ERATS) programs, including preoperative optimization, minimally invasive surgery, opioid-sparing analgesia, early mobilization, patient engagement, and rehabilitation strategies, have been implemented as part of integrated care pathways for lung surgery, aimed at reducing surgical stress and improving functional recovery. Increasing adherence to isolated perioperative care pathway components is associated with a reduction in length of hospital stay, consistent with the ‘marginal gains’ paradigm, whereby the cumulative implementation of multiple ERAS elements confers greater benefit than any single intervention alone. However, evidence-based studies mainly focus on single interventions throughout the perioperative care pathway, and the synergistic effect remains inadequately studied yet. As, individual classic components of ERATS pathways are under development, and innovative versions emerge, this review summarizes contemporary developments across the pre-, intra-, and postoperative phases of lung cancer surgery, focusing on prehabilitation, three-dimensional (3D) imaging, uniportal robot-assisted thoracoscopic surgery (uRATS), non-intubated video-assisted thoracoscopic surgery (NI-VATS), locoregional anesthesia techniques, and drainless surgery. Furthermore, we explore their potential synergistic role in optimizing recovery after lung cancer surgery.

## 1 Introduction

Lung cancer is the leading cause of cancer-related mortality worldwide (1). For patients with early-stage disease, surgical resection remains the treatment of choice with curative intent. Over the past decades, perioperative care in lung cancer surgery has undergone substantial transformation. Enhanced recovery after surgery (ERAS) programs, originally established in colorectal and gastrointestinal surgery, have since been translated into thoracic surgery, giving rise to thoracic-specific perioperative care principles, collectively referred to as enhanced recovery after thoracic surgery (ERATS). These ERATS pathways integrate preoperative optimization, minimally invasive surgery, opioid-sparing analgesia, early mobilization, patient engagement, and rehabilitation strategies. In clinical practice, these approaches are increasingly implemented as part of an integrated care pathway for lung surgery aimed at reducing surgical stress and improving functional recovery and patient-centered outcomes.

Despite the intended shift towards integrated perioperative care for lung cancer surgery patients in recent years, evidence-based studies mainly focus on single interventions throughout the perioperative care pathway. Current guidelines predominantly describe the effects of individual components, rather than the effects of combined perioperative strategies as used in routine practice (2). A recent study on ERATS, however, showed that when adherence to perioperative pathway components increases, the length of stay decreases, which is in line with the “marginal gains” philosophy that application of multiple ERAS-elements produces greater benefit than individual interventions (3). In addition, perioperative innovations may interact with other pathway components in a positive synergetic way, accelerating improvement in outcomes. However, this synergistic effect remains inadequately studied yet. Meanwhile, individual classic components of ERATS pathways are under development, and innovative versions emerge, possibly leading to further enhancement of outcomes, as compared to their older versions.

This narrative review summarizes contemporary developments across the pre-, intra-, and postoperative phases of lung cancer surgery, focusing on prehabilitation, three-dimensional (3D) imaging, uniportal robot-assisted thoracoscopic surgery (uRATS), non-intubated video-assisted thoracoscopic surgery (NI-VATS), locoregional anesthesia techniques, and drainless surgery. By synthesizing the current developments, we aim to provide a comprehensive overview of modern perioperative strategies to discuss their clinical outcomes, workflow challenges, and patient-selection, and explore their potential synergistic role in optimizing recovery after lung cancer surgery.

## 2 Preoperative innovations

### 2.1 Prehabilitation

Prehabilitation aims to enhance functional reserve so that patients are better equipped to withstand the physiological stress of surgery and return to baseline fitness faster. As functional capacity is linked to surgical complications, this approach may improve clinical outcomes (4). Its importance is endorsed by the European Society for Thoracic Surgery (ESTS) and the ERAS Society in the first European guideline on enhanced recovery after lung surgery (2).

Rather than allowing patients to passively wait for surgery, prehabilitation reframes the preoperative period as an active phase of preparation. This structured program, preferably multimodal, is delivered in the two to four weeks between diagnosis and surgery and is already widely used in other fields, such as colorectal surgery (5, 6). Currently, it is becoming increasingly adopted in lung cancer care. It typically combines supervised (high-intensity) exercise training, addressing both aerobic fitness and muscle strength, nutritional optimization, respiratory physiotherapy, psychological support, and smoking cessation (7, 8).

Recent systematic reviews and randomized controlled trials demonstrate that prehabilitation prior to lung cancer surgery can reduce postoperative complications, shorten length of hospital stay (LOHS), and improve functional recovery (9–11). Improvements in aerobic capacity and pulmonary function are particularly relevant in thoracic surgery, where respiratory complications remain a leading cause of morbidity. Furthermore, patients undergoing prehabilitation may report enhanced quality of life, although these effects have been inconsistently reported between studies (12). Important omissions in quality or availability of evidence are based on heterogeneity between programs, a lack of knowledge on minimal/optimal effective duration, effect on oncological delay, patient selection, and cost-effectiveness. Effect estimates of individual components suggest that exercise prehabilitation, nutritional prehabilitation, and multicomponent interventions appear to provide the greatest benefits (13). Also, psychological prehabilitation appears to be effective in reducing postoperative complications (14).

While the clinical impact of prehabilitation may be beneficial and its implementation has proven feasible (12), it still presents logistical challenges due to the narrow timeframe between diagnosis and surgery. Hence, coordinated pathways are required to integrate expertise from multiple disciplines, including physiotherapists, dieticians, and psychologists, to carefully plan its implementation to avoid delays in surgical scheduling (15). Furthermore, as prehabilitation can be delivered through different models, each offer distinct advantages but are also associated with certain limitations. For example, in-person training programs demand significant resources, such as specialized staff and facilities, which may strain hospital capacity. Conversely, digital and hybrid models offer scalable alternatives, delivering exercise regimens, nutritional counseling, and psychological support remotely, although compliance is more challenging (16, 17). The choice of model therefore requires careful consideration at the institutional level, balancing available resources, patient needs, and feasibility.

As recommended in the ESTS guidelines, the potential gain of prehabilitation is most pronounced in patients at elevated risk of postoperative complications, such as those with reduced pulmonary reserve, frailty, or comorbidities (2). Likewise, patients with a lower baseline 6-minute walk distance (< 400 meter) may derive greater benefit from prehabilitation (18). Completing the program, however, may be more challenging in these patients due to their comorbidities. Therefore, tailoring interventions to individual risk profiles would seem reasonable, ensuring that resources are directed toward patients most likely to benefit. On the other hand, in lung cancer surgery, nearly all patients can be considered high risk due to the inherent physiological demands of thoracic procedures and higher incidence of smoking-related comorbidities. Consequently, universal application of prehabilitation may be justified, with particular emphasis on respiratory training and early mobilization.

Looking ahead, several innovations are poised to reshape prehabilitation in thoracic oncology. Risk stratification, personalized intensity, digital coaching, patient-reported outcome measures (PROMS)-driven monitoring, and integration in ERATS dashboards hold potential to enhance its implementation and effectiveness. Digital platforms will likely expand access, reduce resource burdens, and enable real-time monitoring of patient progress. Personalized programs informed by advanced data models and body composition analysis could refine patient selection and optimize intervention intensity. Emerging technologies such as virtual reality and biofeedback may help regulate perioperative stress, enhancing psychological as well as physical resilience. Moreover, integration of wearable devices could provide continuous feedback on physical activity and recovery metrics, fostering patient engagement and adherence. Within the coming years, prehabilitation may evolve into a highly individualized, technology-driven component of perioperative care, seamlessly integrated into an ERATS pathway.

### 2.2 Three-dimensional imaging

While surgical imaging originally relied on computed tomography (CT), magnetic resonance imaging (MRI), and ultrasonography (19), recent developments have expanded this to include high-fidelity three-dimensional (3D) reconstructions, extended reality (XR) modalities (virtual, augmented, and mixed reality), additive manufacturing techniques such as 3D printing, and artificial intelligence (AI)-based methods for image processing (20, 21). In general, a variety of 3D segmentation techniques, referring to the annotation of anatomical structures across CT-scan slices, have enabled the transformation of two-dimensional (2D) grayscale images into volumetric 3D reconstructions of patient anatomy (22–24). This transition facilitates a more in-depth, immersive (XR), and visually intuitive exploration of the lung anatomy, enhancing the understanding of complex spatial relationships between bronchovascular structures (21). Hence, these advanced 3D imaging solutions have shown to enhance multiple aspects of surgical practice: they can improve team experience, confidence, and satisfaction, while also enabling more personalized surgical planning and improving perioperative patient outcomes.

Multiple studies in recent years have underscored the clinical value of advanced 3D imaging tools for segmentectomy planning, shifting their role from optional to nearly mandatory (23–26). Especially since the 2025 ESMO Guidelines have recommended segmentectomy as the preferred surgical approach for early-stage disease, based on the landmark JCOG0802 (27) and CALGB140503 (28) trials comparing lobectomy with segmentectomy. Regarding the technologies employed, several recent reviews have examined the impact of XR systems and 3D-modeling solutions on workflow integration and perioperative outcomes (21, 29, 30). Notably, a recent Delphi consensus report, together with an ESTS expert consensus on segmentectomy technical standards, advocates for the routine use of 3D lung models to support surgeons in both preoperative planning and intraoperative decision-making (25, 31).

Current literature suggests that the use of advanced 3D imaging in segmentectomy may offer several clinical advantages. Observational and predominantly single-center studies with relatively small cohorts report that improved preoperative visualization of patient-specific anatomy and anatomical variations could reduce the need to convert from minimally invasive to open surgery and decrease the risk of inadvertent vascular or bronchial injury. Moreover, 3D imaging may support more refined surgical planning by helping to achieve adequate oncological resection margins, which facilitates lung-sparing strategies where appropriate (25, 30, 32–35). Additionally, potential intra- and postoperative benefits, such as reduced blood loss, shorter operative times, fewer complications, and shorter hospital stay, have all been described. However, given the predominantly retrospective and heterogeneous nature of the existing evidence, high-quality prospective, multicenter, and randomized studies are needed to substantiate these findings. Several of such trials are currently underway, including the PATCHES study by Zaraca et al., the DRIVATS trial, and the Dutch PulmoVR multicenter clinical trial, which are expected to provide more definitive insights into the true clinical impact of advanced 3D imaging in segmentectomy (22, 36–38).

To integrate (3D) imaging into daily clinical practice, several practical prerequisites must be met. These include high-quality contrast-enhanced CT, as well as access to dedicated software for 3D lung reconstructions, ranging from commercially available platforms to validated open-source solutions, depending on costs, surgical volume, technical expertise, and desired customization (e.g., fully automated segmentation) (23). In addition, a dedicated multidisciplinary team, comprising radiologists, surgeons, and technical physicians, is essential for the generation, interpretation, and clinical application of 3D reconstructions. To fully realize the potential of 3D imaging and prevent delays in preoperative planning, continued educational programs, hands-on workshops, and online interactive modules should be incorporated into clinical practice to help normalize its use (23).

The next major advancement in imaging-assisted lung surgery might be real-time integration of advanced imaging technologies in the operating room. In a recent technological report by Sadeghi et al., the integration of various technologies was hybridized during robotic-assisted lobectomy. More specifically, the authors leveraged real-time AI-based robotic-instrument recognition combined with deformable four-dimensional (4D) models to create a real-time imaging overlay of pre-operatively acquired 3D reconstructions onto the actual surgical scene (39). Similarly, Peek et al. and Salerno et al. presented a method for real-time registration of 3D models onto the surgical scene, but more focused on VATS (40, 41). In line with these recent developments, early publications have demonstrated the feasibility of augmented reality for intraoperative localization of pulmonary nodules, marking an important step toward more intuitive and precision-driven surgical guidance (42).

The greatest clinical impact, though, might be expected from future systems capable of reliably detecting and localizing very small nodules or ground-glass opacities using the least invasive localization techniques and enabling the smallest necessary resection margins. Although these developments are promising, they remain at an early stage of clinical development, and further technical refinement and clinical validation are required before routine implementation can be recommended. Achieving this will require true real-time imaging, combined with automated recognition of anatomical structures and vital elements. Such advances will not only support more precise and safer surgery but also pave the way for intraoperative warning systems that can help prevent errors, enhance situational awareness, and significantly shorten learning curves for both novice and experienced surgeons.

## 3 Intraoperative innovations

One of the major breakthroughs in thoracic surgery is the introduction of minimally invasive surgery for oncologic lung resections. After a period of criticism, in which the difficult learning curve and concerns about oncological outcomes prevailed, multiportal video-assisted thoracoscopic surgery (mVATS) for anatomical resections proved its advantage over thoracotomy in patients with early-stage lung cancer, indicating better postoperative outcomes in terms of pain, recovery time, and oncological outcomes (43). These improvements were attributed to the decrease in surgical trauma by moving from thoracotomy to three or four small incisions without rib spreading. However, mVATS was still associated with neuropathic pain (44). Hence, Gonzalez-Rivas propagated that the reduction of the ports to one single incision (uniportal) would reduce surgical trauma, which propelled widespread implementation of this approach (45). To overcome the technical difficulties and learning curve issues of VATS, robotic technology made its entry into thoracic surgery, with clear technological benefits of the robotic platform compared to VATS (46). Nevertheless, robot-assisted thoracoscopic surgery (RATS) was met with even more skepticism than VATS at the time, not only because of the high costs and an increase in waste generation, but also because the financial burden might prevent adoption by resource-constrained hospitals. Furthermore, for uniportal video-assisted thoracoscopic surgery (uVATS) surgeons, the multiportal RATS (mRATS) setup might have represented a step backward in minimizing surgical trauma.

### 3.1 Uniportal robotic-assisted thoracoscopic surgery

Uniportal RATS represents a modification of the conventional mRATS (47). By integrating robotic articulation with single-incision access, uRATS aims to improve surgical ergonomics and keeps on facilitating complex maneuvers within the confined thoracic cavity. This approach may further reduce surgical trauma while maintaining oncological efficacy.

Due to its recent adaptation in clinical practice, the current body of literature on uRATS remains scarce, especially for segmentectomy, and is primarily consisting of small cohort studies or case reports. Overall, uRATS appears to be a safe technique, demonstrating low conversion rates, acceptable perioperative outcomes, and shorter length of hospital stay, for both lobectomy and segmentectomy (48–51). Furthermore, both postoperative pain scores and analgesic use have shown to be lower in patients undergoing uRATS. Although findings regarding operative duration are inconsistent, the available evidence suggests a general trend toward longer operative times in uRATS compared to mRATS (48, 49, 52, 53).

Robust evidence to support clear patient selection criteria is still limited. However, several case reports describe the feasibility of uRATS in complex procedures, including bronchial sleeve resections and operations performed under extracorporeal membrane oxygenation support (54–56). For segmentectomies, particularly when targeting posterior pulmonary segments, u-RATS offers potential advantages over uVATS, primarily because of its greater maneuverability and enhanced 3D visualization (57). Also, RATS in general suggestively excels in patients with narrow intercostal spaces or severe thoracic adhesions, as the instruments are designed to maximize maneuverability with minimal torque on the chest wall. Also, in obese patients, a thick chest wall creates unfavorable fulcrum effects and limits the reach of standard VATS instruments, which potentially can be overcome by the fixed ports and scaled movements of RATS. Furthermore, patients who require extensive lymph node dissection have been suggested to benefit from robotic approaches (51, 58, 59).

Whereas uRATS may offer combined benefits of robotics and a uniportal approach, there are some drawbacks. Not only do surgeons need to be proficient in uVATS and mRATS, but the learning curve, the collisions between the instruments, the lack of the benefit of the third instrument, and maybe even the use of a 0° instead of a 30° endoscope, are currently limiting the approach to a small number of centers. For the implementation of this technique, proper team-training, including training for crisis management are imperative.

The future of uRATS, and maybe even RATS in general, is likely to be shaped by ongoing technological refinement, such as improved haptic feedback, instrument flexibility, and imaging integration. These advances are expected to further enhance surgical precision and facilitate increasingly complex resections. However, with the emergence of such new robotic platforms, such as the Single Port™, da Vinci 5™, and the Ion Endoluminal System™ (60) robotic navigation bronchoscopic platform, robust studies on safety, feasibility, and potential superiority between approaches remain essential.

### 3.2 Non-intubated video-assisted thoracoscopic surgery

While the previously described minimally invasive techniques have been shown to reduce surgical trauma, they require conventional anesthetic strategies. The traditional anesthetic approach necessitated by thoracoscopic surgery has introduced its own challenges, including ventilator-induced lung injury, an increased risk of postoperative pulmonary complications, and potential laryngotracheal trauma associated with the use of double-lumen tubes and bronchial blockers (61). To mitigate these complications, thoracic surgeons and anesthesiologists collaboratively developed the NI-VATS strategy, in which a lung operation is performed while maintaining spontaneous ventilation (62).

The primary advantage of NI-VATS lies in the preservation of native respiratory physiology. Maintaining spontaneous ventilation minimizes perioperative lung injury by preventing alveolar overdistension and subsequent barotrauma (63). Furthermore, preserved diaphragmatic function reduces atelectasis, improves hemodynamic stability, and optimizes ventilation-perfusion ratio, collectively mitigating the risk of developing postoperative pulmonary complications (64, 65). Spontaneous ventilation has also been associated with an attenuated systemic inflammatory and neuroendocrine response, preserving immune function and improving tolerance to adjuvant chemotherapy, with potential long-term oncological benefits (66, 67).

Clinical outcomes of NI-VATS demonstrate lower postoperative pain scores, shortened duration of chest tube drainage, and reduced LOHS (65, 68, 69). Beyond these clinical metrics, NI-VATS offers significant advantages in workflow efficiency. By avoiding complex airway management, such as placement of double-lumen tubes and verification of their position, anesthesia induction time is significantly reduced. Moreover, the surgical preparation is streamlined; patients can actively assist in their own positioning prior to initiation of sedation, ensuring optimal alignment and pressure point protection more rapidly than in an unconscious state. Additionally, carefully titrated sedation and analgesia, typically consisting of propofol and short-acting opioids such as remifentanil or alfentanil, enable a faster emergence compared to general anesthesia, which facilitates accelerated discharge from the post-anesthesia care unit (69). Collectively, these factors reduce total operative and anesthesia times, thereby optimizing operating room efficiency.

To identify suitable patients for NI-VATS anatomical lung resection, a multidisciplinary preoperative assessment is essential, ensuring consensus on surgical and anesthetic feasibility. Technical feasibility in a spontaneously breathing patient must be established, while the anesthesiologist assesses suitability for deep sedation and the accessibility for locoregional techniques by considering the following contraindications: patients with an anticipated difficult airway, increased aspiration risk, persistent cough, excessive airway secretions, (morbid) obesity (BMI > 30–35 kg/m^2^), and severe pre-existing cardiopulmonary disease (i.e., FEV_1_ < 60% predicted, DLCO < 50% predicted, and predicted postoperative FEV_1_ < 30%) (68, 70). Furthermore, the collective expertise of the operative team is crucial for the success and safety of NI-VATS. The surgeon must be experienced in managing a dynamic surgical field, particularly during segmentectomy where mediastinal movement may be more prominent, while the anesthesiologist requires proficiency in both thoracic locoregional techniques and emergent conversion to one-lung ventilation. Clear conversion protocols are therefore essential (63), as conversion to one-lung ventilation rates occurs in approximately 2.8 to 11% of anatomical resections (71), whereas conversion rates during wedge resection are generally lower (72). Conversion becomes mandatory in cases of severe physiological deterioration (i.e., refractory hypoxemia, uncontrollable hypercapnia) or adverse surgical conditions (i.e., major bleeding, dense pleural adhesions, severe mediastinal movements) (68).

NI-VATS remains in an early developmental phase and is currently limited to specialized centers. Hence, within the following years, it is possible that it could become the standard of care for clinically appropriate non-anatomical resections, while more complex anatomical lung resections will continue to require an individualized approach. Future progress depends on improved patient selection and well-defined conversion protocols to ensure safety. Technological advances, including AI, may further support perioperative decision-making. At the same time, a combination of non-intubated anesthesia with RATS has scarcely been described (73), but remains technically demanding, with limited evidence to support its broader adoption, and its future will likely depend on overcoming challenges related to intraoperative airway control and patient stability within the constraints of robotic surgery.

## 4 Postoperative recovery facilitation

### 4.1 Locoregional anesthesia

Thoracic epidural analgesia (TEA) has historically been the standard regional analgesic technique in thoracic surgery. Although TEA provides excellent pain control when correctly placed, related side-effects such as hypotension, immobility, and anxiety before placement are not compliant with ERATS principles. Hence, locoregional anesthetic techniques have gained increasing attention in recent years as alternative to TEA. These peripheral nerve blocks, including thoracic paravertebral block (PVB), erector spinae plane block (ESPB), serratus anterior plane block (SAPB), and intercostal nerve block (ICNB), target specific nerves supplying the chest wall, providing effective analgesia while avoiding the systemic effects associated with epidural blockade. By preserving hemodynamic stability and facilitating early mobilization, peripheral nerve blocks are considered more compatible with ERATS pathways. This is supported by the American Society of Anesthesiologists practice guideline, published in 2026, which recommends peripheral nerve blocks as part of a multimodal analgesic regimen after cardiothoracic surgery (74). Similarly, the PROSPECT guidelines (2021) advise against TEA, even not as second-line option after VATS, due to the associated adverse effects, which compromise mobility (75).

Many studies have investigated the clinical outcomes of peripheral nerve blocks as locoregional analgesic technique, but high-quality evidence has only recently emerged. In 2025, three randomized controlled trials provided robust evidence on these techniques following VATS (76–78). As such, ESPB has shown to be non-inferior to TEA in terms of quality of recovery, but TEA was associated with lower pain scores and significantly less use of intravenous opioids. In this comparative study, LOHS was equal between TEA and ESPB (76). Another study investigating PVB and ICNB in comparison to TEA has shown that ICNB was non-inferior to TEA regarding pain, while PVB was inconclusive. However, both ICNB and PVB were associated with significantly less opioid consumption and demonstrated significantly increased postoperative mobility and decreased postoperative nausea and vomiting (PONV). Also, LOHS was shorter in patients with ICNB but not with PVB (78). Collectively, these findings support the role of peripheral nerve blocks as effective alternatives to TEA in patients undergoing VATS. In the pursuit of optimal locoregional analgesia, ICNB has demonstrated superior efficacy compared with ESP block, resulting in significantly lower morphine consumption, reduced pain scores, and a decreased need for rescue analgesia. However, no differences were found regarding LOHS (77).

Whereas these RCTs primarily focus on analgesia-related outcomes such as pain, quality of recovery, or morphine use, the broader advantages of the less-invasive locoregional techniques lie within the ERATS pathways (e.g., earlier mobility, less PONV, shorter LOHS). Therefore, the choice of analgesic technique should follow a patient-centered approach, in which patients should be informed about advantages and disadvantages of all possible analgesic approaches. A patient preference study showed that most patients undergoing VATS lung surgery find the state of awareness (under general analgesia instead of awake) and enhanced postoperative mobility to be the most important factors regarding postoperative pain (79). Notably, 92% of patients are willing to accept more frequent periods of pain in exchange for the benefits of locoregional analgesia and enhanced postoperative recovery.

Appropriate patient selection remains a key determinant of successful implementation of innovative perioperative strategies. While accumulating evidence supports less invasive approaches, the generalizability of these findings to higher-risk populations remains uncertain. This is particularly relevant for patients with severely impaired pulmonary reserve or those undergoing more extensive procedures, in whom the balance between the benefits and potential limitations of less invasive perioperative interventions may differ. Similarly, the role of adjunctive techniques such as cryoanalgesia may differ in these patients, as its potential benefits appear to be most pronounced in patients undergoing thoracotomy, whereas its added value in minimally invasive procedures seems less suitable. Future studies should specifically evaluate these high-risk subgroups to better define which patients are most likely to benefit from alternative perioperative analgesic strategies and whether recommendations derived from lower-risk populations can be safely extrapolated.

From a workflow perspective, the choice of analgesic technique may impact operating room efficiency. TEA is typically performed preoperatively by the anesthesiologist, whereas other techniques, such as ICNB, are often performed intraoperatively by the surgeon, potentially prolonging operative time. In addition, variability in expertise and the lack of standardized protocol may limit consistent implementation of locoregional strategies. Consequently, both logistical considerations and team experience, next to patient preferences, should be considered when selecting the optimal analgesic approach.

Hence, the future of locoregional analgesia will likely focus on optimizing technique selection and standardized protocols in line with ERATS and patient preferences to maximize analgesic efficacy while minimizing variability in clinical outcomes.

### 4.2 Drainless lung surgery

The current standard practice after anatomical lung resection is to place a chest drain to evacuate air and pleural fluid to prevent lung collapse. According to recent ESTS and American Society of Thoracic Surgery (STS) guidelines, digital drainage systems with a water seal, corresponding to passive suction between -2 cmH_2_O and -8 cmH_2_O, are preferred over traditional analogue drainage systems and active suction (2, 80). While conferring clinical benefit, chest drain placement is also associated with several drawbacks, including a prolonged hospital stay with associated higher healthcare costs, reduced mobility, and patient discomfort (81). Aiming to enhance recovery after lung surgery, progressive chest tube management and flexible drain removal criteria have been implemented using ERATS protocols. With the implementation of digital drainage systems, air leak trends can now be assessed over time rather than relying solely on isolated measurements. As such, thresholds for air leak and fluid output have been raised to <40 mL/min and <450 mL/day, respectively. Notably, even the omission of a fluid output threshold has been investigated and shown to be feasible in selected patients (82). Furthermore, drain removal is increasingly encouraged on postoperative day (POD) 0 in the absence of an air leak (83, 84).

Nowadays, it is believed that it is not always necessary to place a chest tube after wedge resection except in patients with bullous and/or emphysematous lung changes, and that unnecessary placement of a chest tube delays recovery. Multiple studies have already demonstrated that omitting chest drains (85–87), or performing on-table drain removal (88) after wedge resection is associated with a shorter length of hospital stay and reduced postoperative pain. However, findings regarding chest drain reinsertion rates remain inconsistent. While a recent systematic review and meta-analysis reported higher reinsertion or thoracocentesis rates with a risk ratio of 3.02 (86), several individual studies found no significant difference in reinsertion rate compared to standard drainage protocols (85, 87, 88). Omitting chest drains after anatomical lung resection, such as lobectomy or segmentectomy, has not yet been widely reported. The potential risk of serious complications, including tension pneumothorax, continues to deter many surgeons from adopting a drainless approach after lobectomy. Only a few retrospective studies have investigated chest drain omission for anatomical lung resection in carefully selected cases (89–92). Although specific criteria (e.g., patient characteristics) for being eligible for drain omission were not clearly defined and the sample sizes were limited, the results of these studies are encouraging, showing low complication and reinsertion rates, shorter length of hospital stay, and reduced patient discomfort.

In principle, the feasibility of drainless surgery after both wedge resection and anatomical lung resection depends on strict adherence to predefined intraoperative criteria. The absence of air leak is considered essential and has primarily been assessed using digital drainage systems (89, 91). If, in these studies, the air leak is below a predefined threshold value (i.e., 0mL/min), the drain was removed at the end of the surgery before the patient leaves the operation room. However, especially for lobectomy and other anatomical resections, the current evidence is limited. The absence of an evidence-based threshold for acceptable chest drain reinsertion rates is an important consideration. Further research is required to define the most optimal, patient-tailored chest drain management strategy in the perioperative setting, including the role of drainless surgery as a potential option in carefully selected patients. Importantly, future studies should establish safety benchmarks, acceptable chest drain reinsertion rates, and standardized contingency protocols for postoperative complications. Refinement of ERATS protocols and objective air-leak assessment may allow broader implementation of drain omission strategies. Continued prospective evaluation and standardized selection criteria will be essential to ensure patient safety as these care pathways evolve.

Despite ongoing advances in perioperative care and drain management, it is expected that a subset of patients will continue to require postoperative chest drainage due to factors such as intraoperative air leak or compromised lung quality. Nevertheless, the refinement of ERATS protocols, combined with objective intraoperative and immediate postoperative air leak assessment using digital drainage systems, may allow further expansion of drain omission strategies. In the future, patients without perioperative air leakage may safely undergo thoracic surgery without routine chest drain placement, potentially enabling treatment in a same-day discharge surgery or short-stay setting.

## 5 Synergy between perioperative innovations and its future

Despite the rapid expansion of perioperative innovations in lung cancer surgery, these techniques are currently most often studied as stand-alone interventions. This reductionist approach overlooks the inherent interdependence of perioperative care components, in which decisions and interventions applied in one phase shape the effectiveness and outcomes of subsequent phases. A systematic, integrated evidence-based approach encompassing the entire perioperative care pathway and emerging techniques as described in this review is thus still lacking. As a result, potential synergies between preoperative optimization, intraoperative techniques, and postoperative recovery facilitating strategies may remain underexploited.

Emerging evidence from ERATS programs suggests that adherence to the overall pathway may be more important than any individual component. Recent data from the Dutch national ERATS trial demonstrated that higher ERATS adherence was associated with shorter length of stay and fewer postoperative complications after lung resection, supporting the concept that perioperative innovations should increasingly be evaluated as integrated care bundles rather than isolated interventions (3). Future research should therefore move beyond single-intervention studies and focus on integrated perioperative care models as often implemented in daily clinical practice. Developing and evaluating comprehensive, multidisciplinary perioperative care pathways may allow individual interventions to mutually reinforce each other, leading to more consistent, efficient, and patient-centered recovery trajectories after lung cancer surgery.

Within the overarching goal of patient-tailored care, with the ERATS principles serving as a guiding framework, and recognizing the benefits of integrated interventions over isolated approaches, we have conceptualized potential synergistic interactions between perioperative strategies. Prehabilitation is one of the isolated innovations in perioperative care which may work synergistically with other strategies of perioperative care. It is usually incorporated in an ERATS pathway and a continuum with postoperative early mobilization and rehabilitation, and as such has established benefits in perioperative care in general, and in conventional intubated VATS. However, its potential to facilitate broader deployment in NI-VATS requires further elucidation (11). Hypothetically, these programs may expand the eligibility for NI-VATS by optimizing cardiopulmonary reserve of high-risk patients who may derive the greatest clinical benefit from avoiding the physiological stressors associated with endotracheal intubation and positive pressure ventilation. This potential is supported by the literature, showing a lower incidence of pulmonary complications in patients with interstitial lung disease undergoing NI-VATS (93, 94).

In parallel, 3D imaging may represent another synergistic innovation for safety and feasibility of NI-VATS and minimal invasive surgery in general. As discussed in the NI-VATS section, minimizing the risk of intraoperative conversion is paramount. By enhancing patient-specific anatomical understanding, 3D imaging may facilitate operative planning and reduce intraoperative uncertainty, thereby potentially lowering conversion rates to thoracotomy (33). Moreover, improved intraoperative decision-making enabled by 3D imaging could reduce operation time and influence downstream postoperative strategy. One such potential synergistic application is the combination of 3D imaging with drainless anatomical lung resection. Because the feasibility of drainless surgery is largely determined by postoperative air leak from lung parenchyma, 3D imaging may help prevent parenchymal injury through more precise dissection and planning.

Furthermore, the combination of uRATS with locoregional anesthetic techniques may represent a synergistic approach to reduce overall perioperative burden. The limited surgical trauma associated with a uniportal approach may potentiate the effectiveness of peripheral nerve blocks by minimizing nociceptive input. In this context, the alignment of reduced tissue disruption and targeted analgesia may facilitate improved pain control, earlier mobilization, and faster recovery.

Looking ahead, future research should make the next step and move beyond the study of isolated interventions and instead focus on the integration of multiple promising elements within bundled, pathway-based strategies, as intended in an ERATS program. More specific, future studies should focus on a dose-response relationship between ERATS adherence and outcomes, instead of the evaluation of before-and-after effects of the implementation of isolated ERATS components. Such studies can possibly give more insight into which component most strongly drives outcomes such as recovery and LOHS, while highlighting the interdependence of ERATS elements, given that isolated components may not show measurable effects when evaluated in isolation. Furthermore, it remains largely unknown whether combining perioperative innovations results in merely additive effects or whether meaningful synergistic interactions exist. Addressing this question should be a priority for future research, particularly as increasingly complex ERATS pathways are being adopted in clinical practice despite limited evidence regarding the contribution and interaction of individual components. A better understanding of these relationships may help ensure that pathway development remains evidence-driven rather than based on the cumulative implementation of promising but insufficiently validated interventions. The overall goal should thus be to study these components as integrated elements of a care pathway to facilitate the development of optimally patient-tailored perioperative programs and at the same time, reduce unwarranted practice variation that is based on lack of guideline implementation or coordination. Such an approach enables perioperative care to better account for patient heterogeneity. It should be supported by outcome frameworks that extend beyond traditional measures of functional recovery to incorporate patient-reported outcomes, thereby providing a more comprehensive and patient-centered evaluation of recovery. Ideally, continuous outcome monitoring on a dashboard should be available to show real-time postoperative outcomes of the perioperative pathway. New, promising, innovative interventions could then be introduced in a stepwise fashion into the already existing pathway, allowing assessment of their effects on the postoperative outcomes and thereby generating evidence of its contribution to ERATS.

It should be acknowledged, though that studying an ERATS program is inherently challenging, precisely due to its multimodal and bundled design, as observed associations may reflect confounding by indication or reverse causality. Introduction of a new innovative intervention into the perioperative pathway should ideally be preceded by research showing at least a trend of positive effects of the specific intervention on postoperative outcome, to prevent antagonistic interactions with other components. Moreover, this complexity may be further compounded by secular trends. In addition, a significant evidence gap remains regarding the individual effectiveness and cost-effectiveness of many emerging perioperative innovations. This important aspect falls beyond the scope of the present narrative review but should be addressed in future research.

### 5.1 Artificial intelligence

In this rapidly evolving high-technology era, and with the increasing integration of AI in healthcare, it is likely that AI will play a significant role in the next step towards patient-tailored ERATS care, besides its role in the variety of isolated ERATS aspects. In the ideal scenario, a patient’s visit to the outpatient clinic would be supported by a program that generates a personalized treatment pathway integrating patient-specific characteristics and individual preferences. At the push of a button, individualized treatment strategies can be generated. However, the implementation of such approaches is not without challenges, particularly regarding patient privacy and security, and the responsible use of sensitive medical data. At present, predictive models are developed to identify patients at risk for complications, using patient demographics, surgical details, and postoperative recovery data (95). Such models may also support patient selection for specific treatment strategies, for example by predicting the risk of failure of NI-VATS or identifying who benefits most from prehabilitation programs. In addition, AI-assisted 3D reconstruction can be combined with augmented reality to provide interoperative anatomical navigation, supporting complex minimally invasive resections (42). Moreover, AI is increasingly important in diagnosing molecular (sub)types of cancer, which should be considered in individualized treatment strategies.

Despite the many potential benefits of the development and implementation of AI in lung cancer care and in medicine more broadly, the use of AI is accompanied by substantial costs. To date, no published data are available on cost effectiveness of AI in lung cancer surgery. However, a recently published study showed that the clinical implementation of AI-based prediction models for decision support in colorectal cancer surgery can be a cost-effective strategy (96). Nevertheless, direct extrapolation of these findings to lung cancer surgery is challenging due to differences in procedural characteristics, perioperative workflows, and resource utilization. Future research is needed to translate these findings to lung cancer surgery.

## 6 Conclusion

Innovations in perioperative lung cancer care are rapidly evolving, with promising results, aiding in enhanced recovery. The next advancement lies in integrating isolated components into a multimodal, bundled, and patient-tailored ERATS program. By integrating patient-specific preferences and characteristics, and leveraging synergies between individual perioperative components, truly personalized treatment strategies can be formed, shaping the future of lung cancer care.

## Glossary

ERAS: enhanced recovery after surgery
ERATS: enhanced recovery after thoracic surgery
3D: three-dimensional
uRATS: uniportal robot assisted thoracoscopic surgery
NI-VATS: non-intubated video assisted thoracoscopic surgery
ESTS: European Society for Thoracic Surgery
LOHS: length of hospital stay
PROMS: patient reported outcomes measures
CT: computer tomography
MRI: magnetic resonance imaging
XR: extended reality
AI: artificial intelligence
2D: two-dimensional
ESMO: European Society For Medical Oncology
4D: four-dimensional
mVATS: multiportal video assisted thoracoscopic surgery
uVATS: uniportal video assisted thoracoscopic surgery
mRATS: multiportal robot assisted thoracoscopic surgery
TEA: Thoracic epidural analgesia
PVB: paravertebral block
ESPB: erector spinae plane block
SAPB: serratus anterior plane block
ICNB: intercostal nerve block
PONV: postoperative nausea and vomiting
STS: Society of Thoracic Surgery
POD: postoperative day;
